# Wastewater analysis of chemical markers of public health concern at small spatial scales: A scoping review

**DOI:** 10.1371/journal.pgph.0005058

**Published:** 2025-09-10

**Authors:** Oxana Klassen, Susanne Moebus, Dennis Schmiege

**Affiliations:** Institute for Urban Public Health (InUPH), University Hospital Essen, University of Duisburg-Essen, Essen, Germany; Universidad de Colima, MEXICO

## Abstract

Wastewater analysis is a promising approach to obtaining population-based health information. It has proven useful for different applications, including monitoring illicit drugs or assessing population-level exposure to chemicals. Studies have often analysed samples from wastewater treatment plants, which does not allow for small-scale intra-sewershed differentiations needed for a detailed assessment of the target population. The small-scale approach offers various benefits, but a comprehensive review of its application to chemicals has not yet been undertaken. This scoping review aims to provide a detailed overview of the current knowledge on wastewater analysis of chemical markers of public health concern, including methodological aspects. We conducted a systematic database search for peer-reviewed articles. Data were analysed using quantitative summaries and qualitative narrative synthesis. Out of 2283 articles, 99 studies were included. Most were published after 2010. The studies analysed wastewater from different settings, with a focus on points of interest such as healthcare and education facilities, and few studies at neighbourhood-level. Pharmaceuticals, industrial and environmental chemicals and stimulants were most commonly investigated. While most studies reported their sampling mode, few provided detailed specifications. Small-scale sampling sites were characterized to varying degrees, with many studies reporting only a single characterization criterion. Advancing the area of research of small-scale wastewater analysis requires consistent and transparent methodological reporting and a more detailed sampling site characterization. Wastewater analysis of chemical markers of public health concern at small spatial scales shows great potential for further public health applications, including occupational health and monitoring chemical exposure in schools.

## 1. Introduction

The analysis of biological and chemical markers of human activity (hereafter referred to as biomarkers) [1] in wastewater is a promising approach to obtaining health and disease information at the population level [2]. This area of research is sometimes referred to as “wastewater-based epidemiology” (WBE). While there is no single definition of WBE, it can be described as “the scientific field of linking pathogens and chemicals found in wastewater to population-level health” [3]. First formulated conceptually in 2001 [4], the interest in WBE and its applications has grown considerably over the past two decades.

WBE leverages the fact that people excrete biomarkers in urine and faeces. This sewage is often collected in a sewer system, allowing the analysis of these biomarkers within geographically defined sewersheds. Such wastewater analyses can provide valuable population-level insights into illicit drug use [5–9], the circulation of pathogens [10,11] and antimicrobial resistance [12–14] or exposure to toxicants [15,16]. WBE has some advantages over traditional epidemiologic survey methods, with shortcomings such as self-reporting and low response rates [17]. It could provide anonymous and near real-time estimates of lifestyle and disease indicators in a cost-effective manner compared to testing a similar number of people. WBE also has certain limitations [18], such as the unclear number of contributing populations and variable excretion rates [19].

Previous studies analysed wastewater often at the wastewater treatment plant (WWTP) level. While this is sufficient for many applications, it does not allow for small-scale intra-sewershed differentiations. Small-scale WBE offers additional applications such as hotspot identification [20] source tracking [21] or, due to its higher sensitivity compared to the WWTP level, even earlier detection of biomarkers in wastewater as opposed to clinical surveillance systems [22]. In this study, we use “small-scale” to refer to studies that analysed wastewater from within the sewer system, i.e., not exclusively at the inlet of a WWTP, with no absolute population or area size cut-off.

Small-scale WBE is particularly relevant for large sewersheds in urban and metropolitan areas that can serve several hundreds of thousands of people. Within the sewer system, wastewater can be sampled to represent either an area of interest (AOI), such as neighbourhoods [6,23], or a points of interest (POI), such as education [24–26] or healthcare facilities [27–29].

Despite the many advantages of small-scale WBE applications, wastewater sampling in the sewer system requires additional considerations. While evidence was synthesized for the application of small-scale WBE to infectious diseases and antibiotic resistance [30], there is no comprehensive overview to date for chemical markers of public health concern. Focusing on these chemicals is particularly important because exposure data are often scarce, especially at small scales, while these substances are often ubiquitous in the environment and potentially harmful to humans. For example, endocrine disrupting chemicals such as plasticizers or pesticides are linked to a range of adverse health effects in humans, but are often not sufficiently regulated, resulting in ongoing exposure [31,32]. Analysing parent compounds, i.e., “the starting compound in a biotransformation reaction” [33], and metabolites, i.e., “the product of a biotransformation reaction” [34] of such harmful chemicals in wastewater might be a promising approach to reveal population-level exposures and identify communities or specific sub-groups at increased risk.

This scoping review aims to contribute to filling the information gap by systematizing the existing knowledge on the application of small-scale WBE in the context of chemical markers of public health concern, thereby highlighting existing research gaps, methodological considerations and areas for future research. Specifically, we examined (a) the chemical biomarkers analysed in wastewater, (b) the settings (spatial scale), (c) the objectives pursued and (d) the details of the sampling procedure, including criteria for the selection of the small-scale sampling points.

## 2. Materials and methods

### 2.1 Search strategy and selection procedure

This scoping review aimed to identify peer-reviewed literature [35] that analysed wastewater within a sewer system, focusing either on a specific AOI or POI. We systematically searched three scientific databases: PubMed, Scopus and Web of Science (last search date: 12.06.2025). The search used three sets of indexed and free-text terms in various combinations: (i) wastewater or sewage, (ii) surveillance, monitoring, or epidemiology, and (iii) a list of terms related to small-scale approaches, including specific building types (all search terms are listed in Table A in S1 Text). The reporting adheres to the PRISMA (Preferred Reporting Items for Systematic reviews and Meta-Analyses) statement [36] and its guidelines for scoping reviews [37].

The literature search had no year or language restriction. Only original research articles that focus on chemical compounds of public health concern were included, without any further restrictions on the (bio)markers analysed. Studies were excluded if they (a) sampled wastewater only at the inlet or outlet of a WWTP; (b) focused exclusively on the development or validation of analytical methods; (c) were written in languages other than English; (d) were not original research articles, e.g., conference papers, abstracts, book chapters, reviews, opinion pieces or letters to the editor (the full list of inclusion and exclusion criteria can be found in Table B).

We used a multi-step approach to identify relevant studies. After the initial database search, titles and abstracts of the records were screened and entries were removed according to the inclusion and exclusion criteria. In case of uncertainty, the record was retained for full text analysis. Studies were then assessed for eligibility using the full text. In addition, the reference section of each included article was screened to potentially capture studies not identified in the initial search. Disagreements during study selection and data extraction were resolved through discussion, and, if needed, with the involvement of a third reviewer.

### 2.2 Data items, collection process and synthesis methods

Two authors (OK and DS) carried out the data extraction from full text articles individually. A customized data extraction Google Spreadsheet was used, facilitating the collaboration and integration of data. The collected data included: (i) details about the studies such as authors, title, year, journal, article type, location; (ii) the spatial scale of the study; (iii) information about the wastewater biomarkers; (iv) the objectives of each study; (v) details about the sampling process, including timing and type, number of samples and criteria for selecting sampling points; and (vi) specific actions implemented based on the wastewater analysis (a full overview of the data items can be found in Table C in S1 Text). As this scoping review aims to provide a comprehensive thematic overview of the application of small-scale WBE to chemicals of public health concern, no quality assessment of the included articles was performed.

Extracted data were analysed using both quantitative summaries and qualitative narrative synthesis. While some data items, e.g., year or number of samples, were straightforward to analyse, others, e.g., geographic location, spatial scale, biomarkers and sampling methods were grouped for a clearer overview. Free text fields, such as study objectives and interventions were examined using an iterative process. Keywords that captured the essence of each entry were derived, refined and categorized into broader themes.

For the spatial scale, we distinguished between POIs and AOIs. POIs were further categorized into functional groups, such as “education” or “healthcare”. Each functional group was further subdivided into specific settings, where applicable. For example, “education” was subdivided into “university” and “school”, whereas “healthcare” was subdivided into “hospital” and “long-term care facility”, among others (Table D in S1 Text). The spatial scale, including functional groups and specific settings, was also used to stratify other data items, e.g., chemical biomarkers and information on the sampling procedure.

In order to obtain an overview of the chemical biomarkers tested, all studies were screened for the biomarkers, these were noted and then grouped into logically related groups. The biomarkers that were assigned to “pharmaceuticals” were then subdivided again according to Anatomical Therapeutic Chemical (ATC) Classification.

## 3. Results

### 3.1 Study selection

**Fig 1:**
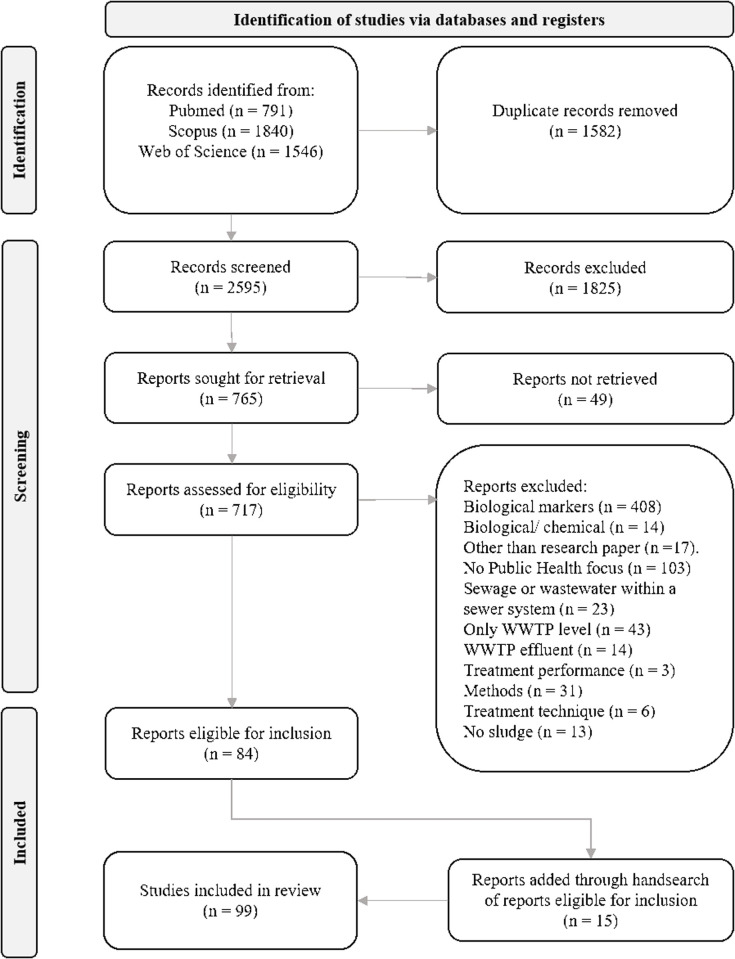
PRISMA flow chart of the scoping review showing the identification and selection of relevant studies [36].

Fig 1 shows the PRISMA flow chart of identifying and selecting studies for final inclusion. The initial search in the three scientific databases yielded 3865 articles. After duplicate removal and screening of the titles and abstracts, 765 reports remained for retrieval. For articles that were behind a paywall, we contacted the respective corresponding authors. It was not possible to obtain 48 articles, leaving 717 articles for the assessment of eligibility. After applying the inclusion and exclusion criteria, 84 articles were eligible for inclusion. A hand search of the included studies resulted in additional 15 articles suitable for inclusion. Ultimately, our scoping review included 99 studies (the full list of all studies included can be found in S1 Text).

### 3.2 Study characteristics

Since the first publication in 1996, the number of publications has increased exponentially over the last decades, suggesting a growing interest in small-scale WBE (Graph in S1 Text). A total of 36 countries were identified in the 99 articles (Table E in S1 Text), revealing an uneven global distribution. Many studies were conducted in Northern America (19/99; 19%), Western (17; 17%) or Southern Europe (16; 16%) and Eastern Asia (12; 12%), while only eleven studies were conducted in different African sub-regions. No studies were found for Australia and New Zealand, Central Asia or Oceania. Applying the World Bank’s Income Classification, most studies (58; 59%) were conducted in high-income countries (Table F in S1 Text), followed by upper (20; 20%) and lower-middle income countries (21; 21%). No studies were conducted in low income countries.

### 3.3 Synthesis of the included studies

#### 3.3.1 *Overview of study objectives.*

Using a qualitative approach, by extracting keywords from the respective objective paragraph(s) of each included study, different types of objectives were identified. Studies focused on (i) the detection, identification or measurement of specific chemical biomarkers (57/99; 58%) or (ii) on the assessment, estimation, evaluation or prediction of chemical compounds in wastewater (68; 69%). In addition, some focused in parts (and not exclusively) on (iii) the development, optimization and/or validation of analytical methods (24; 24%). The majority of the studies were assigned to a single objective group, with a small number of studies addressing several objectives (Table G in S1 Text).

#### 3.3.2 *Spatial scale and settings.*

**Fig 2:**
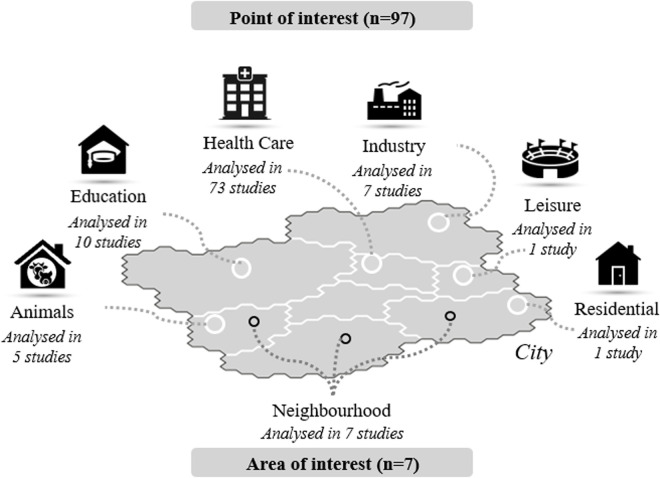
Number of studies analysing wastewater at areas or points of interests in different settings.

Within the sewer system, wastewater can be sampled to cover two spatial scales (Table D in S1 Text). Fig 2 provides an overview of all settings found in the studies. Only few studies focused on AOIs, i.e., neighbourhoods (7/99; 7%), while the majority of studies examined wastewater from POIs (92; 93%). POIs were further categorized into different functional groups: Healthcare (73/92; 79%), encompassing hospitals and nursing and care facilities, education (10; 11%), comprising universities and schools, industry (7; 7%), including industries and companies, animals (5; 5%), referring to livestock farms and their wastewater treatment facilities, leisure (1; 1%), in this case a football stadium, and residential (1; 1%).

Most studies focused on one spatial scale and a single functional group, with two exceptions where wastewater was sampled from „Neighbourhood“ and “Healthcare” [12] and “Neighbourhood“ and “Industry” [38]. Some studies focused on several buildings of the same functional group, e.g., hospital and retirement homes [12], hospitals, nursing and assisted and independent living care [39–41] or hospital and long-term care facility [29].

The majority of studies sampled one small-scale site (43/99; 43%), followed by sampling between two and up to five (28; 28%), between six and nine (13; 13%) or between twelve and 20 sites (8; 8%). A few studies sampled more than 20 small-scale sites (7; 7%) (Table H in S1 Text). The number of small-scale sites sampled in the studies varied between the spatial scales (Table I in S1 Text). On average (median), neighbourhood studies sampled six small-scale sites (min: 1; max: 15), while POI-focused studies analysed, on average, wastewater from two sites (min: 1 max: 39). Differences were also observable between POI-focused studies regarding the functional groups (Table I in S1 Text). The average (median) number of small-scale sites sampled was seven for animals (min: 1 max: 27), one for education (min: 1 max: 1), two for healthcare (min: 1 max: 39) and eight for industry (min: 5 max: 27).

#### 3.3.3 *Chemical biomarkers in wastewater.*

**Fig 3:**
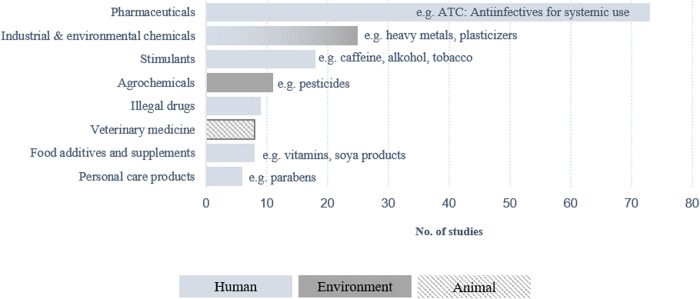
Chemical biomarkers with examples analysed in the included studies, grouped by human, environment and animal domains.

A variety of chemical biomarkers was analysed in the studies. They were categorized into the following groups according to their main application: pharmaceuticals (77/99; 77%), industrial and environmental chemicals (28; 28%), stimulants (20; 20%), agrochemicals (13; 13%), food additives and supplements (10; 10%), illicit drugs (10; 10%), veterinary medicine (8; 8%) and personal care products (7; 7%). (Fig 3 and Table J in S1 Text). Most applications were grouped in the human domain, followed by environment and animal. One application could be classified to both human and environmental.

The “pharmaceuticals” as the largest group was further subdivided according to the ATC classification [42]. In total, 14 generic categories could be derived (Table K in S1 Text). Pharmaceuticals from the category (N) Nervous system, such as painkillers or antidepressants (44/75; 59%) was tested most frequently, followed by (J) Antiinfectives for systemic use, such as antibiotics and antifungals (42; 56%) and (C) Cardiovascular system, such as beta blockers for heart therapy (40; 53%). The markers were examined or found to varying degrees in the seven different settings.

Pharmaceuticals were analysed most frequently in healthcare (61/77; 79%), industrial & environmental chemicals were analysed most commonly in healthcare (14/28; 50%). Stimulants were analysed most frequently in healthcare (14/20; 70%). Agrochemicals were analysed most commonly in healthcare (5/13; 38%) and neighbourhood (4/13; 31%). Food additives and supplements were analysed most frequently in healthcare (7/10; 70%). Illicit drugs were analysed most frequently in healthcare (4/10; 40%) and education (3/10; 30%). Veterinary medicine were analysed most commonly in animals (4/8; 50%) and healthcare (4/8; 50%). Personal Care Products were analysed most commonly in healthcare (5/7; 71%). (Table L in S1 Text).

#### 3.3.4 *Sampling at smaller spatial scales and characterization of sub-sewersheds.*

The majority of studies (59/99; 60%) reported the number of wastewater samples analysed (Table M in S1 Text). On average (median), 32 wastewater samples were analysed in the studies, with a wide range between one and 851. In the first quintile, studies (13/59) analysed between fewer than or equal to ten wastewater samples, while in the highest quintile studies (12/59) analysed equal to or more than 68 samples. Few studies analysed a very high number of samples, with 140 [43], 156 [44], 322 [45], 568 [46] or 851 wastewater samples [47].

**Fig 4:**
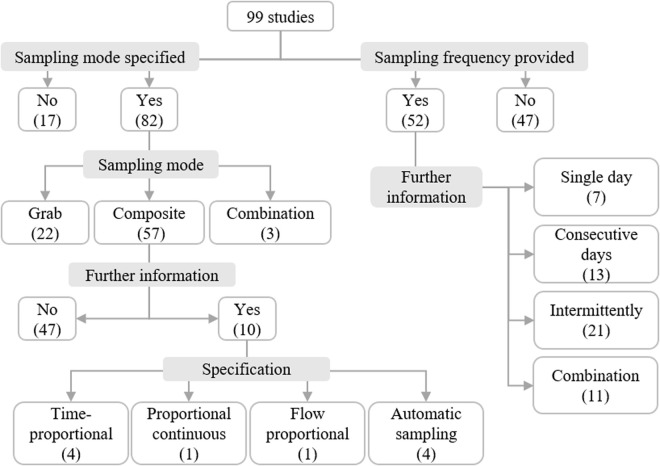
Sampling mode specified and sampling frequency provided.

Most studies reported on their sampling mode (Fig 4; Table N in S1 Text). Composite sampling was utilized most, followed by (individual) grab sampling. The majority of studies employed one sampling mode, while three studies combined them. Only very few studies provided further specified their sampling mode. The sampling frequency could be derived in 52 studies (Fig 4). Some studies sampled wastewater on consecutive days, while others sampled intermittently, and a few took a sample on only a single day. In addition, eleven studies employed a combination of intermittent and consecutive sampling (Table O in S1 Text). Many studies in this scoping review did not provide any details about their sampling frequency.

Additional information on the specific characteristics of the sampling sites was compiled from the included studies, hereafter referred to as “characterization criteria”. Small-scale sampling sites were characterized in most studies (83/99, 83%), but to varying degrees, while 16 studies did not provide further information on their sampling sites. A single characterization criterion was reported in 52 studies, while 31 studies reported multiple criteria.

Due to the predominance of studies conducted in the healthcare setting, we considered these separately from the other settings in terms of characterization criteria (see Table P in S1 Text). Of those studies that provided additional information in the functional group “healthcare” (66), healthcare facilities were characterized by geographic location (27/66, 41%), name (16/66, 24%), capacity - indicated by number of beds (17/66, 26%), specialization (14/66, 21%), departments (13/66, 20%) and their level of pharmaceutical use (3/66, 5%). Studies not belonging to the functional group “healthcare” used the following characterization criteria: location (10), name (6), type of sampling point (3), accessibility (1), land use (1) or willingness to participate (1). In AOI, hardly any information was provided on the characterization criteria; in individual cases, the criterion “land use” was mentioned in the neighbourhoods.

## 4. Discussion

### 4.1 Standardized reporting of sampling increases transparency and reproducibility

Biomarker concentrations and wastewater discharge show greater variation at small-scale sampling sites compared to aggregated units such as a WWTP [12,27,48–53]. Accurately contextualizing the results of small-scale WBE studies therefore requires detailed information on the sampling procedure, as sampling modes and intervals significantly affect the results, e.g., the representativeness of the sample, and interpretation of chemical compounds in wastewater [54].

The lack of transparency in reporting of sampling makes it difficult to contextualize the results of the study or to assess the potential impact of methodological limitations on the results and conclusions drawn. The provision of a minimum of essential information, such as the exact number of samples, the sampling mode (e.g., grab vs. composite sampling) with further specifications (e.g., sampling mode, 24-hour or time of day) is desirable to assess the potential sources of variability and uncertainty [54,55] arising from the sampling process. Developing a standardized reporting framework could lead to greater transparency, facilitating comparisons between studies and enhancing the reproducibility of small-scale WBE research studies.

### 4.2 Detailed understanding of the local conditions leads to more accurate interpretations

A comprehensive understanding of the geographical context in small-scale WBE studies is essential. Identification and quantification of the different sources of chemical compounds is warranted, as concentrations may vary depending on the source. For example, the presence of a major emitter of a particular chemical compound will result in spatial variations between sampling sites that are not necessarily associated with differences in human exposure. A detailed characterization of the sewer system, including its sewershed and the specific conditions at the small-scale sampling point is essential and requires information beyond the simple identification of the sewer system or the name of the small-scale sampling point.

Sewersheds, i.e., geographical areas where wastewater flows to a single endpoint [56], may differ in land use patterns, population, and environmental characteristics, which, in turn, may affect the occurrence of the chemical of interest. For example, a sewershed that includes large areas of agricultural land may exhibit higher concentrations of pesticides or fertilizers in the wastewater, potentially reflecting surface runoff from nearby fields. In contrast, an urban sewershed with dense residential or commercial land use may show elevated levels of pharmaceuticals and personal care products. In addition, if, for example, there are differences in the metabolic rates of a certain chemical substance, e.g., by sex or age, the back-calculation could be improved if the number of women and men or age (groups) is available for the sewershed. However, arriving at such population characteristics proves difficult, as there is often a mismatch between sewershed and administrative boundaries.

To address this effectively, Geographic Information Systems (GIS) are a valuable tool in small-scale WBE research [57], as they can play an important role in thoroughly characterizing the sewershed and site-specific conditions. GIS can be used to overlay and analyse different spatial datasets, including sewer system infrastructure, sewershed boundaries, land use, administrative boundaries with population data and geo-referenced point sources. For example, by overlaying small-scale sampling locations with land use data, it is possible to identify areas with a large number of industrial facilities or areas with high agricultural activity, which may be associated with elevated concentrations of certain pollutants in wastewater. The combination of these data layers can be used to create detailed maps that provide insight into the relationships between potentially influencing elements and their impact on the occurrence of chemical biomarkers in wastewater [58]. The integration of spatial data improves the understanding of local conditions and can lead to a more accurate interpretation of small-scale WBE results.

### 4.3 Ethical considerations in small-scale WBE

Although it is generally not possible to trace wastewater analysis back to an individual [59], the analysis of wastewater from small-scale sampling points, such as schools or workplaces, requires careful consideration of ethical aspects to reflect on the implications associated with the investigation. For example, the publication of high rates of drug use in a school could lead to the stigmatization of students, whether or not they are actually taking the drug [17], while in working environments, the discovery of drug residues could increase mistrust on the part of employers, which can even result in dismissals [17]. The unintended damage described illustrates the need for careful and sensitive handling of the results of small-scale wastewater analyses. Applying professional ethical guidelines in small-scale WBE research helps to minimize potential unintended harm and ensure scientific honesty [59].

### 4.4 Potential fields of research in small-scale WBE

WBE could prove to be a powerful tool for obtaining population-based health information, with a lot of untapped potential. For example, the analysis of wastewater from specific settings or locations within a community could provide valuable insights about the health and disease status of a specific population group. This could help create a more detailed picture of the health challenges faced by different segments of the community and enables the development of targeted public health interventions, as outlined by the examples in the following.

The use of WBE in occupational health and safety is a promising but largely untapped area of research. By analysing wastewater discharged from industrial or large corporate sites, it is possible to gain valuable insights into the chemical substances to which people are exposed during their work [60,61]. This approach would offer certain advantages over traditional occupational health monitoring methods. First, WBE could provide a comprehensive overview of the total chemical burden in a workplace because it captures exposures from multiple sources, including production processes or cleaning agents. Second, it could enable the identification of emerging contaminants that may not have been previously recognized as occupational hazards. This information could be used to develop targeted preventive measures, such as improving ventilation systems, introducing personal protective equipment, or modifying production processes. By identifying occupational exposures, WBE could lead to significant reductions in occupational disease, disability, and mortality, thereby contributing to a healthier and more productive workforce.

A potential area of research in small-scale WBE could be to conduct analyses in settings where children and adolescents spend a considerable amount of time, such as kindergartens, day-care centres and schools. These institutions serve as key places of children’s lives, providing opportunities to investigate a range of potential exposures. Childhood is an important developmental period during which children are particularly susceptible to harmful influences [62]. Health disruptions in this early life stage can have long-term consequences for health and well-being in adulthood [62]. In this context, endocrine disrupting chemicals (EDC), which can disrupt the hormone system, require particular attention as they can increase the risk of diseases across the lifespan [62]. The adverse health effects of these toxic compounds may not be noticed immediately or shortly after exposure. Phthalates, for instance, can are linked to obesity in (preschool) children [63,64] and can also impair lung function [65]. In addition to phthalates, children are exposed to numerous other environmental chemicals that can be found in air, water, house dust, toys, clothing, furniture and food [66]. WBE may become an important tool for assessing the children’s exposure to EDCs by enabling the non-invasive and cost-effective monitoring of chemicals that enter the child’s body and are potentially harmful to their health. Particularly with regard to children’s health, WBE could help to track population-level trends in exposure to chemicals or identify novel or emerging contaminants. These initial results could be followed up with human biomonitoring studies to confirm the presence of such emerging contaminations. Based on these results, appropriate preventive measures could be developed as a first step towards a safe and healthy environment for children’s health and development.

### 4.5 Limitations

Only peer-reviewed studies published in English were included, which results in the exclusion of relevant grey literature or research published in other languages. Furthermore, in line with the methodological framework of scoping reviews, no formal assessment of study quality was conducted. Consequently, variations in the methodological rigor and reporting quality of the included studies may occur, but these should not affect the summarizing nature of this scoping review.

## 5. Conclusion

This scoping review highlights the growing interest in and potential of small-scale wastewater-based epidemiology (WBE) for monitoring chemical markers of public health concern. Small-scale WBE was used in 99 studies in a variety of settings, focusing mainly on points of interest, e.g., healthcare or education facilities, rather than areas of interest, such as neighbourhoods. In addition, a wide range of chemical markers were analysed, with pharmaceuticals being the most common, followed by industrial & environmental chemicals and stimulants, demonstrating its broad applicability for analysing small-scale chemical exposure. Our results also reveal considerable variation in the reporting of sampling and the characterization of the small-scale sampling sites.

Reporting of key aspects of sampling was insufficient in many studies, highlighting the need for standardized reporting in small-scale WBE studies. In addition, only few studies comprehensively characterized their small-scale sampling sites. Accurate contextualization of data, including transparent reporting of sampling technique, mode and frequency, as well as key characteristics of the sampling site, is essential for minimizing methodological uncertainty and ensure that results are robust and reproducible.

Small-scale WBE holds promise for exploring different areas of public health, such as occupational health and monitoring exposure in institutions frequented by children, such as day-care centres and schools. The ability to detect emerging contaminants and assess the cumulative chemical burden in specific population groups could provide invaluable insights into health risks.

In conclusion, while small-scale WBE is a promising tool for analysing chemical marker of public health concern, there is a pressing need for consistent and transparent reporting of methodological aspects, and further research into its applications in low-resource settings. As the field continues to evolve, interdisciplinary collaborations and the development of standardized protocols will be crucial in unlocking the full potential of small-scale WBE to enhance our understanding of chemical exposures and their impacts on public health.

## Supporting information

S1 Text (DOCX)
