# Supplementary material for: Wastewater analysis of chemical markers of public health concern at small spatial scales: A scoping review
Source: PLOS Glob Public Health. 2025 Sep 10;5(9):e0005058. doi: 10.1371/journal.pgph.0005058 (PMC12422432; doi:10.1371/journal.pgph.0005058)
Supplement: S1 Text — (DOCX) [file pgph.0005058.s001.docx]

**Supplementary Information for:**

**Wastewater analysis of chemical markers of public health concern at small spatial scales: A scoping review**

**Authors:** Klassen, Oxana^a*^, Moebus, Susanne^a^, Schmiege, Dennis^a,^

^a^ Institute for Urban Public Health (InUPH), University Hospital Essen, University of Duisburg-Essen, 45130 Essen, Germany.

* Corresponding author:

Oxana Klassen

Institute for Urban Public Health, University Hospital Essen

Hufelandstr. 55, 45147 Essen, Germany

E-Mail: [oxana.klassen@uk-essen.de](mailto:oxana.klassen@uk-essen.de)

ORCID: <https://orcid.org/0009-0005-0239-3770>

Susanne Moebus:

ORCID: <https://orcid.org/0000-0002-0072-5410>

Dennis Schmiege:

ORCID: <https://orcid.org/0000-0002-4450-9680>

Table of Content

[Tables – Overview 3](#_Toc184291791)

[References - All Studies included 16](#_Toc184291792)

[Figures 23](#_Toc184291793)

# Tables – Overview

[Table A: Search terms 2](#_Toc184285905)

[Table B: Inclusion and exclusion criteria 2](#_Toc184285906)

[Table C: Overview of the variables 3](#_Toc184285907)

[Table D: Functional groups and their specific settings 4](#_Toc184285908)

[Table E: WBE and countries 5](#_Toc184285909)

[Table F: Income countries 5](#_Toc184285910)

[Table G: Objective overview 6](#_Toc184285911)

[Table H: SS sampled 6](#_Toc184285912)

[Table I: SS sampled and their functional groups 6](#_Toc184285913)

[Table J: Functional groups of chemical markers and their examples 6](#_Toc184285914)

[Table K: Medicine and ATC 7](#_Toc184285915)

[Table L: Chemical markers and their functional groups 8](#_Toc184285916)

[Table M: Number of samples 8](#_Toc184285917)

[Table N: Sampling type 8](#_Toc184285918)

[Table O: Sampling frequency 9](#_Toc184285919)

[Table P: Overview characterization criteria 9](#_Toc184285920)

Table A: Search terms

| Keywords | Search terms |
| --- | --- |
| Keywords (in title) | (sewage OR wastewater OR waste water OR “environment* surveillance”)  AND  (small-scale OR “small scale” OR  sub-sewershed* OR subsewershed* OR  municipal OR community OR residential OR  neighborhood* OR  building* OR  school* OR university OR college OR campus OR  hospital* OR nursing OR healthcare OR “health care” OR decentralized OR upstream) |
| Keywords (in abstract) | (surveillance OR monitoring OR tracing OR tracking OR epidemiology) |

Table B: Inclusion and exclusion criteria

| Criteria | Description |
| --- | --- |
| Inclusion criteria | - Studies analysing sewage or wastewater within a sewer system, can either be at an area or point of interest - No year restriction - No restriction on (bio)marker analysed - Different article types: research, review, commentary, opinion, letter to the editor - Studies with a clear public health focus (human health) |
| Exclusion criteria | - Studies sampling wastewater only at the treatment plant level - Studies investigating (WWTP) effluent - Studies examining only treatment performance - Other languages than English and German - No conference papers, abstracts, presentations, book chapters - Development of analytical methods - Development of treatment techniques - Studies focusing exclusively on effluents - Focus on sludge - No editorial |

Table C: Overview of the variables

| Variable-Name | Explanation/ Definition |
| --- | --- |
| ID (new) | The ID is individual for each publication and helps to better assign the information |
| First author | The first named author of the publication |
| Title | Title of the Publikation |
| Year | Year of Publikation |
| Journal | Journal of Publikation |
| Article type | Article Type of Publikation |
| Location | The country or city in which the study was conducted. |
| Spatial scale | The spatial level that has been sampled. Either points of interest (POI), in the form of a building (e.g. hospital, care facility, university, industry) or area of interest (AOI) in the form of a neighborhood. |
| Marker | Chemical markers |
| Marker specified | Markers are subdivided and defined into functional groups such as: agrochemicals, food additives and supplements, illicit drugs, industrial and environmental chemicals, personal care products, pharmaceuticals, stimulants, veterinary drugs |
| Objective(s) | The objectives found in the studies, evaluated qualitatively afterwards |
| Sampling period | Period in which the sampling took place |
| Number of samples | Number of samples taken in the study |
| WWTP(s) sampled | Number of wastewater treatment plants that have been sampled |
| SS sampled | Number of spatial scales that have been sampled |
| Selection criteria (or name) | qualitative analysis |
| Sampling (site, time, type) | qualitative analysis |
| Interventions | qualitative analysis |
| Spatial findings | qualitative analysis |
| Further remarks_DS | qualitative analysis |
| Further remarks_OK | qualitative analysis |

Table D: Functional groups and their specific settings

| **spatial-scale** | **functional group** | **number of studies** | **(specific) setting** | **absolute** | **relative (%)** |
| --- | --- | --- | --- | --- | --- |
| POI | Animals | 5 | swine, poultry and dairy farms | 2 | 40 |
|  |  |  | concentrated animal feeding operation sites | 1 | 20 |
|  |  |  | animal waste treatment facilities | 1 | 20 |
|  |  |  | livestock | 1 | 20 |
| POI | Education | 10 | university | 9 | 90 |
|  |  |  | school | 1 | 10 |
| POI | Health Care | 73 | hospitals | 62 | 85 |
|  |  |  | nursing care | 4 | 5 |
|  |  |  | assisted and independent living | 4 | 5 |
|  |  |  | health care facility | 1 | 1 |
|  |  |  | long-term care facility | 1 | 1 |
|  |  |  | retirement home | 1 | 1 |
| POI | Industry | 7 | industry | 5 | 71 |
|  |  |  | enterprise | 1 | 14 |
|  |  |  | beauty salon | 1 | 14 |
| POI | Leisure | 1 | football stadium | 1 | 100 |
| POI | Residental | 1 | residental | 1 | 100 |
| AOI | Neighbourhood | 7 | neighbourhood | 7 | 100 |

| **Continent** | **M49 sub-region** | **# countries** | **% countries** | **% continent** | **# studies** | **% studies** | **% continent** |
| --- | --- | --- | --- | --- | --- | --- | --- |
| Africa | Southern Africa | 1 | 3% | 17% | 1 | 1% | 11% |
|  | Middle Africa | 1 | 3% |  | 1 | 1% |  |
|  | Western Africa | 2 | 6% |  | 2 | 2% |  |
|  | Northern Africa | 1 | 3% |  | 4 | 4% |  |
|  | Eastern Africa | 1 | 3% |  | 3 | 3% | 23% |
| Americas | Northern America | 2 | 6% | 14% | 19 | 19% |  |
|  | Central America | 2 | 6% |  | 3 | 3% |  |
|  | South America | 1 | 3% |  | 1 | 1% | 30% |
| Asia | Eastern Asia | 3 | 8% | 39% | 12 | 12% |  |
|  | South-eastern Asia | 4 | 11% |  | 7 | 7% |  |
|  | Southern Asia | 5 | 14% |  | 7 | 7% |  |
|  | Western Asia | 2 | 6% |  | 4 | 4% | 35% |
| Europe | Northern Europe | 1 | 3% | 31% | 1 | 1% |  |
|  | Eastern Europe | 1 | 3% |  | 1 | 1% |  |
|  | Southern Europe | 5 | 14% |  | 16 | 16% |  |
|  | Western Europe | 4 | 11% |  | 17 | 17% | 100% |
|  |  | 36 |  | 100% | 99 | 100% |  |
|  |  |  |  | NAs | 0 |  |  |
|  |  |  |  | all | 99 |  |  |

Table E: WBE and countries

Table F: Income countries

| **Income** | **# countries** | **% countries** | **# studies** | **% studies** |
| --- | --- | --- | --- | --- |
| HIC | 17 | 47% | 58 | 59% |
| UMIC | 8 | 22% | 20 | 20% |
| LMIC | 11 | 31% | 21 | 21% |
|  | 36 | 100% | 99 | 100% |

Table G: Objective overview

| Groups | Absolute | Relative (in %) |
| --- | --- | --- |
| Objectives I | 57 | 58 |
| Objectives II | 24 | 24 |
| Objectives III | 68 | 69 |

Table H: SS sampled

| SS sampled (groups) | absolute | Relative (in %) |
| --- | --- | --- |
| 1 | 43 | 43 |
| 2-5 | 28 | 28 |
| 6-9 | 13 | 13 |
| 10-20 | 8 | 8 |
| More than 20 | 7 | 7 |

Table I: SS sampled and their functional groups

| SS sampled (groups) | Total | point of interest | | | | | | | | | | | | area of interest | |
| --- | --- | --- | --- | --- | --- | --- | --- | --- | --- | --- | --- | --- | --- | --- | --- |
|  |  | Animals | | Education | | Health | | Industry | | leisure | | residental | | Neighbourhood | |
|  |  | abs. | rel. | abs. | rel. | abs. | rel. | abs. | rel. | abs. | rel. | abs. | rel. | abs. | rel. |
| 1 | 43 | 1 | 20 | 8 | 80 | 31 | 45 | 0 | 0 | 1 | 100 | 0 | 0 | 2 | 29 |
| 2-5 | 28 | 0 | 0 | 2 | 20 | 2 3 | 33 | 1 | 14 | 0 | 0 | 1 | 100 | 1 | 14 |
| 6-9 | 14 | 2 | 40 | 0 | 0 | 6 | 9 | 4 | 57 | 0 | 0 | 0 | 0 | 1 | 14 |
| 10-20 | 10 | 0 | 0 | 0 | 0 | 6 | 9 | 1 | 14 | 0 | 0 | 0 | 0 | 3 | 42 |
| more than 20 | 6 | 2 | 40 | 0 | 0 | 3 | 4 | 1 | 14 | 0 | 0 | 0 | 0 | 0 | 0 |

Table J: Functional groups of chemical markers and their examples

| Groups of Chemical Markers | examples (excerpted) |
| --- | --- |
| agrochemicals | Pesticides |
| food additives and supplements | Vitamins, soya products, wound healing |
| illegal drugs | Illegal drugs |
| industrial & environmental chemicals | Heavy metals, plasticizers, dyes, surfactants |
| Personal Care Products | Parabens, UV protectants |
| medicine | Medicine, see ATC |
| stimulants | Caffeine, alcohol, tobacco |

Table K: Medicine and ATC

| **ATC_short** | **ATC_Groups** | **category** | Points of Interest | | | | | | | Area of Interest |
| --- | --- | --- | --- | --- | --- | --- | --- | --- | --- | --- |
|  |  |  | total | Animal | Education | Health Care | Industry | Leisure | residental | Neighborhood |
| A | Alimentary tract and metabolism | medicine | 30 | 2 | 1 | 25 | 0 | 1 | 0 | 1 |
| B | Blood and blood forming organs | medicine | 7 | 2 | 0 | 5 | 0 | 0 | 0 | 0 |
| C | **Cardiovascular system** | medicine | 40 | 0 | 1 | 38 | 0 | 0 | 0 | 1 |
| D | Dermatologicals | medicine | 20 | 2 | 3 | 15 | 0 | 0 | 0 | 0 |
| G | Genito urinary system and sex hormones | medicine | 19 | 0 | 1 | 18 | 0 | 0 | 0 | 0 |
| H | Systemic hormonal preparations, excl. sex hormones and insulins | medicine | 2 | 0 | 2 | 0 | 0 | 0 | 0 | 0 |
| J | **Antiinfectives for systemic use** | medicine | 42 | 3 | 1 | 38 | 0 | 0 | 0 | 0 |
| L | Antineoplastic and immunomodulating agents | medicine | 18 | 0 | 1 | 17 | 0 | 0 | 0 | 0 |
| M | Musculo-skeletal system | medicine | 20 | 0 | 1 | 18 | 0 | 0 | 0 | 1 |
| N | **Nervous system** | medicine | 44 | 0 | 6 | 36 | 0 | 0 | 0 | 2 |
| P | Antiparasitic products, insecticides and repellents | medicine | 4 | 2 | 0 | 2 | 0 | 0 | 0 | 0 |
| R | Respiratory system | medicine | 17 | 0 | 4 | 13 | 0 | 0 | 0 | 0 |
| S | Sensory organs | medicine | 33 | 2 | 1 | 29 | 0 | 0 | 0 | 1 |
| V | Various | medicine | 8 | 0 | 1 | 7 | 0 | 0 | 0 | 0 |

| Chemical markers | Total | point of interest | | | | | | | | | | | | area of interest | |
| --- | --- | --- | --- | --- | --- | --- | --- | --- | --- | --- | --- | --- | --- | --- | --- |
|  |  | Animals | | Education | | Health Care | | Industry | | leisure | | residental | | Neighborhood | |
|  |  | abs. | rel. | abs. | rel. | abs. | rel. | abs. | rel. | abs. | rel. | abs. | rel. | abs. | rel. |
| agrochemicals | 13 | 1 | 8 | 2 | 15 | 5 | 38 | 1 | 8 | 0 | 0 | 0 | 0 | 4 | 31 |
| food additives and supplements | 10 | 1 | 10 | 1 | 1 | 7 | 70 | 0 | 0 | 0 | 0 | 1 | 10 | 0 | 0 |
| illegal drugs | 10 | 0 | 0 | 3 | 3 | 4 | 40 | 0 | 0 | 1 | 10 | 0 | 0 | 2 | 20 |
| industrial & environmental chemicals | 28 | 0 | 0 | 3 | 11 | 14 | 50 | 7 | 25 | 0 | 0 | 1 | 4 | 4 | 14 |
| Personal Care Products | 7 | 0 | 0 | 2 | 29 | 5 | 71 | 0 | 0 | 0 | 0 | 0 | 0 | 0 | 0 |
| pharmaceuticals | 77 | 5 | 6 | 6 | 8 | 61 | 79 | 0 | 0 | 1 | 10 | 1 | 1 | 3 | 4 |
| stimulants | 20 | 0 | 0 | 4 | 2 | 14 | 70 | 0 | 0 | 0 | 0 | 1 | 5 | 1 | 5 |
| veterinary medicine | 8 | 4 | 50 | 0 | 0 | 4 | 50 | 0 | 0 | 0 | 0 | 0 | 0 | 0 | 0 |

Table L: Chemical markers and their functional groups

Table M: Number of samples

| ID (new) | First author | Title | Year | Number of samples |
| --- | --- | --- | --- | --- |
| Afsa_2020_occurence | Afsa et al. | Occurrence of 40 pharmaceutically active compounds in hospital and urban wastewaters and their contribution to Mahdia coastal seawater contamination | 2020 | 46 |
| Ajibola_2022_quechers | Ajibola et al. | QuEChERS-based analysis and ecotoxicological risk of select antibiotics in dumpsite leachates, hospital wastewater and effluent receiving water in Ibadan, Nigeria | 2022 | 18 |
| Alexander_2022_identification | Alexander et al. | Identification of critical control points for antibiotic resistance discharge in sewers | 2022 | 59 |
| Al-Qaim_2015_investigation | Al-Qaim et al. | Investigation of the environmental transport of human pharmaceuticals to surface water: A case study of persistence of pharmaceuticals in effluent of sewage treatment plants and hospitals in Malaysia | 2015 | 103 |
| Arvantii_2023_occurence | Arvaniti et al. | Occurrence of pharmaceuticals in the wastewater of a Greek hospital: Combining consumption data collection and LC-QTOF-MS analysis | 2023 | 20 |
| Arvantii_2023_study | Arvaniti et al. | Study on the Occurrence of Artificial Sweeteners, Parabens, and Other Emerging Contaminants in Hospital Wastewater Using LC-QToF-MS Target Screening Approach | 2023 | 20 |
| Azuma_2016_detection | Azuma et al. | Detection of pharmaceuticals and phytochemicals together with their metabolites in hospital effluents in Japan, and their contribution to sewage treatment plant influents | 2016 | 6 |
| Bailly_2013_calibration | Bailly et al. | Calibration and field evaluation of Polar Organic Chemical Integrative Sampler (POCIS) for monitoring pharmaceuticals in hospital wastewater | 2013 | 4 |
| Ben_2013_occurence | Ben et al. | Occurrence and partition of antibiotics in the liquid and solid phases of swine wastewater from concentrated animal feeding operations in Shandong Province, China | 2013 | 41 |
| Bowes_2023_integrated | Bowes et al. | Integrated multiomic wastewater-based epidemiology can elucidate population-level dietary behaviour and inform public health nutrition assessments | 2023 | 156 |
| Burgard_2013_potential | Burgard et al. | Potential trends in Attention Deficit Hyperactivity Disorder (ADHD) drug use on a college campus: Wastewater analysis of amphetamine and ritalinic acid | 2013 | 9 |
| Choi_2013_comparison | Choi et al. | Comparison of compositional characteristics of amino acids between livestock wastewater and carcass leachate | 2013 | 7 |
| Daouk_2016_dynamics | Daouk et al. | Dynamics of active pharmaceutical ingredients loads in a Swiss university hospital wastewaters and prediction of the related environmental risk for the aquatic ecosystems | 2016 | 140 |
| Endo_2020_rapid | Endo et al. | Rapid Assessment of Opioid Exposure and Treatment in Cities Through Robotic Collection and Chemical Analysis of Wastewater | 2020 | 70 |
| Eslami_2015_occurence | Eslami et al. | Occurrence of non-steroidal anti-inflammatory drugs in Tehran source water, municipal and hospital wastewaters, and their ecotoxicological risk assessment | 2015 | 36 |
| Foppe_2021_analysis | Foppe et al. | Analysis of 39 drugs and metabolites, including 8 glucuronide conjugates, in an upstream wastewater network via HPLC-MS/MS | 2021 | 5 |
| Giuliani_1996_detection | Giuliani et al. | Detection of genotoxic activity in native hospital waste water by the umuC test | 1996 | 851 |
| Gómez_2006_determination | Gómez et al. | Determination of pharmaceuticals of various therapeutic classes by solid-phase extraction and liquid chromatography-tandem mass spectrometry analysis in hospital effluent wastewaters | 2006 | 6 |
| Gönder_2021_detailed | Gönder et al. | Detailed characterization, antibiotic resistance and seasonal variation of hospital wastewater | 2021 | 8 |
| Goswami_2022_occurence | Goswami et al. | Occurrence of Pharmaceutically Active Compounds and Potential Ecological Risks in Wastewater from Hospitals and Receiving Waters in Sri Lanka | 2022 | 34 |
| Goullé_2012_importance | Goullé et al. | Importance of anthropogenic metals in hospital and urban wastewater: Its significance for the environment | 2012 | 116 |
| Gul_2016_1 | Gul et al. | LC–MS-MS Method for Analysis of Opiates in Wastewater During Football Games | 2016 | 35 |
| Gul_2016_2 | Gul et al. | LC–MS-MS Method for Analysis of Opiates in Wastewater During Football Games II | 2016 | 36 |
| Gushgari_2018_tracking | Gushgari et al. | Tracking narcotics consumption at a Southwestern U.S. university campus by wastewater-based epidemiology | 2018 | 80 |
| Hameed2023_Identification | Hameed, N | Identification of the Components of Wastewater from Al-Sadr Teaching Hospital in Basrah, Iraq | 2023 | 6 |
| Hoang2024_pharmaceuticals | Hoang, A | Environmental risk assessment of selected pharmaceuticals in hospital wastewater in nothern Vietnam | 2024 | 21 |
| Jolibois_2002_glutaraldehyde | Jolibois et al. | Glutaraldehyde in hospital wastewater | 2002 | 10 |
| Knoth_2018_determination | Knoth et al. | Determination of the contamination by azole antimycotics in hospital and house sewage - A pilot project for the city of Dresden | 2018 | 568 |
| Kosma_2010_occurence | Kosma et al. | Occurrence and removal of PPCPs in municipal and hospital wastewaters in Greece | 2010 | 32 |
| Kümmerer_1997_biodegradability | Kümmerer et al. | Biodegradability of the anti-tumour agent ifosfamide and its occurrence in hospital effluents and communal sewage | 1997 | 17 |
| Kunnemeyer_2009_speciation | Kunnemeyer et al. | Speciation Analysis of Gadolinium Chelates in Hospital Effluents and Wastewater Treatment Plant Sewage by a Novel HILIC/ICP-MS Method | 2009 | 14 |
| Lemas_2021_estimating | Lemas et al. | Estimating drug consumption during a college sporting event from wastewater using liquid chromatography mass spectrometry | 2021 | 33 |
| Lenz_2005_presence | Lenz et al. | Presence of cancerostatic platinum compounds in hospital wastewater and possible elimination by adsorption to activated sludge | 2005 | 28 |
| Lenz_2007_monitoring | Lenz et al. | Monitoring, removal and risk assessment of cytostatic drugs in hospital wastewater | 2007 | 98 |
| Li_2022_occurence | Li et al. | Occurrence Characteristics and Ecological Risk Assessment of Organophosphorus Compounds in a Wastewater Treatment Plant and Upstream Enterprises | 2022 | 11 |
| Lien_2016_antibiotics | Lien et al. | Antibiotics in wastewater of a rural and an urban hospital before and after wastewater treatment, and the relationship with antibiotic use-a one year study from Vietnam | 2016 | 66 |
| Lin_2014_ketamine | Lin et al. | Ketamine and the metabolite norketamine: Persistence and phototransformation toxicity in hospital wastewater and surface water | 2014 | 36 |
| Liu_2010_analysis | Liu et al. | Analysis of hormone antagonists in clinical and municipal wastewater by isotopic dilution liquid chromatography tandem mass spectrometry | 2010 | 68 |
| Lopez-Herguedas_2023_suspect | Lopez-Herguedas et al. | Suspect Screening of Chemicals in Hospital Wastewaters Using Effect-Directed Analysis Approach as Prioritization Strategy | 2023 | 1 |
| Mazzitelli_2018_evaluation | Mazzitelli et al. | Evaluation of psychiatric hospital wastewater toxicity: what is its impact on aquatic organisms? | 2018 | 5 |
| Mullot_2009_development | Mullot et al. | Development and validation of a sensitive and selective method using GC/MS-MS for quantification of 5-fluorouracil in hospital wastewater | 2009 | 14 |
| Najam2024_Monitoring | Najam, T | Monitoring of university wastewater within the sewage system and its performance evaluation through integrated constructed wetlands | 2024 | 16 |
| Nasri2024_Pharmaceuticals | Nasri, E | Pharmaceuticals and personal care products in Tunisian hospital wastewater: occurrence and environmental risk | 2024 | 21 |
| Negreira_2014_cytostatic | Negreira et al. | Cytostatic drugs and metabolites in municipal and hospital wastewaters in Spain: Filtration, occurrence, and environmental risk | 2014 | 43 |
| Nkansah_2016_characterization | Nkansah et al. | Characterization of Beauty Salon Wastewater from Kwame Nkrumah University of Science and Technology, Kumasi, Ghana, and Its Surrounding Communities | 2016 | 48 |
| Rodríguez-Rodríguez_2023_occurence | Rodríguez-Rodríguez et al. | Occurrence and risk assessment of pharmaceuticals in hospital wastewater in Costa Rica | 2023 | 32 |
| Sá_2022_tracking | Sá et al. | Tracking pollutants in a municipal sewage network impairing the operation of a wastewater treatment plant | 2022 | 24 |
| Saunders_2016_pharmaceutical | Saunders et al | Pharmaceutical concentrations in screened municipal wastewaters in Victoria, British Columbia: A comparison with prescription rates and predicted concentrations | 2016 | 54 |
| Saussereau_2013_determination | Saussereau et al. | Determination of levels of current drugs in hospital and urban wastewater | 2013 | 116 |
| Sharma_2022_direct | Sharma et al. | Direct injection green chromatographic method for simultaneous quantification of amoxicillin and amikacin in maternity hospital wastewater (Sagar, India) | 2022 | 66 |
| Tania_2022_emerging | Tania et al. | Emerging small-scale textile industries in residential areas of Mirpur, Dhaka City, Bangladesh: an assessment of the discharged wastewater quality and potential impacts. Environmental Monitoring and Assessment | 2022 | 24 |
| Van-Hoi_2021_determination | Van Hoi et al. | Determination of Pharmaceutical Residues by UPLC-MS/MS Method: Validation and Application on Surface Water and Hospital Wastewater | 2021 | 11 |
| Vaudreuil_2022_pharmaceutical | Vaudreuil et al. | Pharmaceutical pollution of hospital effluents and municipal wastewaters of Eastern Canada | 2022 | 42 |
| Verlicchi_2012_hospital | Verlicchi et al. | Hospital effluent: Investigation of the concentrations and distribution of pharmaceuticals and environmental risk assessment | 2012 | 16 |
| Verlicchi_2016_predicted | Verlicchi & Zambello | Predicted and measured concentrations of pharmaceuticals in hospital effluents. Examination of the strengths and weaknesses of the two approaches through the analysis of a case study | 2016 | 8 |
| Wang_2021_comparison | Wang et al. | Comparison of tobacco use in a university town and a nearby urban area in China by intensive analysis of wastewater over one year period | 2021 | 322 |
| Wei_2011_veterinary | Wei et al. | Occurrence of veterinary antibiotics in animal wastewater and surface water around farms in Jiangsu Province, China | 2011 | 53 |
| Wei_2012_ciproflaxicin | Wei et al. | Occurrence of Ciprofloxacin, Enrofloxacin, and Florfenicol in Animal Wastewater and Water Resources | 2012 | 53 |
| Yilmaz_2017_characterization | Yilmaz et al. | Characterization and toxicity of hospital wastewaters in Turkey | 2017 | 3 |

Table N: Sampling type

| Sampling type | absolute | Relative (in %) |
| --- | --- | --- |
| Individual grab sampling | 22 | 22 |
| Composite sampling | 57 | 57 |
| Composite & grab sampling | 3 | 3 |

Table O: Sampling frequency

| Sampling frequency | absolute | Relative (in %) |
| --- | --- | --- |
| Consecutive days | 13 | 13 |
| Intermittent samples | 21 | 21 |
| Consecutive & intermittend | 11 | 11 |
| Single day | 7 | 7 |

Table P: Overview characterization criteria

| Characterization Criteria | absolute | absolute (health) | relative (in %) | relative (health) |
| --- | --- | --- | --- | --- |
| location | 37 | 27 | 37 | 73 |
| name | 22 | 16 | 22 | 73 |
| capacity | 18 | 17 | 18 | 94 |
| specialization | 17 | 14 | 17 | 82 |
| departments | 14 | 13 | 14 | 93 |
| type | 4 | 1 | 4 | 25 |
| Level of pharmaceutical use | 3 | 3 | 3 | 100 |
| timing | 3 | 3 | 3 | 100 |
| accessibility | 1 | 0 | 1 | 0 |
| first infection case SARS-CoV-2 | 1 | 1 | 1 | 100 |
| land use | 1 | 0 | 1 | 0 |
| willingness to participate | 1 | 0 | 1 | 0 |
| NA | 15 | 7 | 15 | 47 |

# References - All Studies included

Afsa, S., et al. (2020). "Occurrence of 40 pharmaceutically active compounds in hospital and urban wastewaters and their contribution to Mahdia coastal seawater contamination." Environ Sci Pollut Res Int 27(2): 1941-1955.

Ajibola, A. S., et al. (2022). "QuEChERS-based analysis and ecotoxicological risk of select antibiotics in dumpsite leachates, hospital wastewater and effluent receiving water in Ibadan, Nigeria." J Environ Sci Health A Tox Hazard Subst Environ Eng 57(8): 709-722.

Alexander, J., et al. (2022). "Identification of critical control points for antibiotic resistance discharge in sewers." Sci Total Environ 820: 153186.

Al-Qaim, F., et al. (2015). "Investigation of the Environmental Transport of Human Pharmaceuticals to Surface Water: A Case Study of Persistence of Pharmaceuticals in Effluent of Sewage Treatment Plants and Hospitals in Malaysia." Journal of the Brazilian Chemical Society 26: 1124-1135.

Arvaniti, O. S., et al. (2023). "Occurrence of pharmaceuticals in the wastewater of a Greek hospital: Combining consumption data collection and LC-QTOF-MS analysis." Sci Total Environ 858(Pt 3): 160153.

Arvaniti, O. S., et al. (2023). "Study on the Occurrence of Artificial Sweeteners, Parabens, and Other Emerging Contaminants in Hospital Wastewater Using LC-QToF-MS Target Screening Approach." Water 15(5): 936.

Aydin, S., et al. (2019). "Antibiotics in hospital effluents: occurrence, contribution to urban wastewater, removal in a wastewater treatment plant, and environmental risk assessment." Environ Sci Pollut Res Int 26(1): 544-558.

Azuma, T., et al. (2016). "Detection of pharmaceuticals and phytochemicals together with their metabolites in hospital effluents in Japan, and their contribution to sewage treatment plant influents." Sci Total Environ 548-549: 189-197.

Bailly, E., et al. (2013). "Calibration and field evaluation of Polar Organic Chemical Integrative Sampler (POCIS) for monitoring pharmaceuticals in hospital wastewater." Environ Pollut 174: 100-105.

Beltifa, A., et al. (2020). "Monitoring hospital wastewaters for their probable genotoxicity." J Water Health 18(1): 1-7.

Ben, W., et al. (2013). "Occurrence and partition of antibiotics in the liquid and solid phases of swine wastewater from concentrated animal feeding operations in Shandong Province, China." Environ Sci Process Impacts 15(4): 870-875.

Bendakovská, L., et al. (2016). "Development of ICP-MS and ICP-OES methods for determination of gadolinium in samples related to hospital waste water treatment." Chemical Papers 70.

Bowes, D. A., et al. (2022). "A framework for wastewater sample collection from a sewage cleanout to inform building-scale wastewater-based epidemiology studies." Sci Total Environ 836: 155576.

Bowes, D. A., et al. (2023). "Integrated multiomic wastewater-based epidemiology can elucidate population-level dietary behaviour and inform public health nutrition assessments." Nature Food 4(3): 257-266.

Burgard, D. A., et al. (2013). "Potential trends in Attention Deficit Hyperactivity Disorder (ADHD) drug use on a college campus: wastewater analysis of amphetamine and ritalinic acid." Sci Total Environ 450-451: 242-249.

Castillo Meza, L., et al. (2020). "Detection and removal of biologically active organic micropollutants from hospital wastewater." Sci Total Environ 700: 134469.

Choi, J. W., et al. (2013). "Comparison of compositional characteristics of amino acids between livestock wastewater and carcass leachate." Environ Monit Assess 185(11): 9413-9418.

Daouk, S., et al. (2016). "Dynamics of active pharmaceutical ingredients loads in a Swiss university hospital wastewaters and prediction of the related environmental risk for the aquatic ecosystems." Sci Total Environ 547: 244-253.

Driver, E. M., et al. (2020). "Alcohol, nicotine, and caffeine consumption on a public U.S. university campus determined by wastewater-based epidemiology." Sci Total Environ 727: 138492.

Emmanuel, E., et al. (2005). "Ecotoxicological risk assessment of hospital wastewater: a proposed framework for raw effluents discharging into urban sewer network." Journal of Hazardous Materials 117(1): 1-11.

Endo, N., et al. (2020). "Rapid Assessment of Opioid Exposure and Treatment in Cities Through Robotic Collection and Chemical Analysis of Wastewater." J Med Toxicol 16(2): 195-203.

Eslami, A., et al. (2015). "Occurrence of non-steroidal anti-inflammatory drugs in Tehran source water, municipal and hospital wastewaters, and their ecotoxicological risk assessment." Environ Monit Assess 187(12): 734.

Evans, E. D., et al. (2020). "Longitudinal wastewater sampling in buildings reveals temporal dynamics of metabolites." PLoS Comput Biol 16(6): e1008001.

Foppe, K. S., et al. (2021). "Analysis of 39 drugs and metabolites, including 8 glucuronide conjugates, in an upstream wastewater network via HPLC-MS/MS." J Chromatogr B Analyt Technol Biomed Life Sci 1176: 122747.

Giuliani, F., et al. (1996). "Detection of genotoxic activity in native hospital waste water by the umuC test." Mutation Research/Genetic Toxicology 368(1): 49-57.

Gómez, M. J., et al. (2006). "Determination of pharmaceuticals of various therapeutic classes by solid-phase extraction and liquid chromatography–tandem mass spectrometry analysis in hospital effluent wastewaters." Journal of Chromatography A 1114(2): 224-233.

Gómez-Canela, C., et al. (2014). "Occurrence of cytostatic compounds in hospital effluents and wastewaters, determined by liquid chromatography coupled to high-resolution mass spectrometry." Anal Bioanal Chem 406(16): 3801-3814.

Gönder, Z. B., et al. (2021). "Detailed characterization, antibiotic resistance and seasonal variation of hospital wastewater." Environ Sci Pollut Res Int 28(13): 16380-16393.

Goswami, P., et al. (2022). "Occurrence of Pharmaceutically Active Compounds and Potential Ecological Risks in Wastewater from Hospitals and Receiving Waters in Sri Lanka." Environ Toxicol Chem 41(2): 298-311.

Goullé, J. P., et al. (2012). "Importance of anthropogenic metals in hospital and urban wastewater: its significance for the environment." Bull Environ Contam Toxicol 89(6): 1220-1224.

Gros, M., et al. (2013). "Rapid analysis of multiclass antibiotic residues and some of their metabolites in hospital, urban wastewater and river water by ultra-high-performance liquid chromatography coupled to quadrupole-linear ion trap tandem mass spectrometry." J Chromatogr A 1292: 173-188.

Gu, D., et al. (2019). "Occurrence and Risk Assessment of Antibiotics in Manure, Soil, Wastewater, Groundwater from Livestock and Poultry Farms in Xuzhou, China." Bull Environ Contam Toxicol 103(4): 590-596.

Gul, W., et al. (2016). "LC–MS-MS Method for Analysis of Opiates in Wastewater During Football Games II." Journal of Analytical Toxicology 40(5): 330-337.

Gul, W., et al. (2016). "LC–MS-MS Method for Analysis of Opiates in Wastewater During Football Games II." Journal of Analytical Toxicology 40(5): 330-337.

Gushgari, A. J., et al. (2018). "Tracking narcotics consumption at a Southwestern U.S. university campus by wastewater-based epidemiology." J Hazard Mater 359: 437-444.

Hameed, N. and M. Al-Enazi (2023). "Identification of the Components of Wastewater from Al-Sadr Teaching Hospital in Basrah, Iraq." Egyptian Journal of Aquatic Biology and Fisheries 27: 203-21

Hernandez-Ramírez, A., et al. (2021). "Determination of Pharmaceuticals Discharged in Wastewater from a Public Hospital Using LC-MS/MS Technique." Journal of the Mexican Chemical Society 6

Hoang, A. T. P., et al. (2024). "Environmental risk assessment of selected pharmaceuticals in hospital wastewater in nothern Vietnam." Chemosphere 356: 141973.

Iloms, E., et al. (2020). "Investigating Industrial Effluent Impact on Municipal Wastewater Treatment Plant in Vaal, South Africa." Int J Environ Res Public Health 17(3).

Jolibois, B., et al. (2002). "Glutaraldehyde in Hospital Wastewater." Archives of Environmental Contamination and Toxicology 42(2): 137-144.

Kimosop, S. J., et al. (2016). "Residue levels and discharge loads of antibiotics in wastewater treatment plants (WWTPs), hospital lagoons, and rivers within Lake Victoria Basin, Kenya." Environ Monit Assess 188(9): 532.

Kinuthia, G., et al. (2020). "Levels of heavy metals in wastewater and soil samples from open drainage channels in Nairobi, Kenya: community health implication." Scientific Reports 10: 8434.

Klančar, A., et al. (2016). "Levels of pharmaceuticals in Slovene municipal and hospital wastewaters: a preliminary study." Arh Hig Rada Toksikol 67(2): 106-115.

Kleywegt, S., et al. (2016). "The contribution of pharmaceutically active compounds from healthcare facilities to a receiving sewage treatment plant in Canada." Environ Toxicol Chem 35(4): 850-862.

Knoth, H., et al. (2018). "Determination of the contamination by azole antimycotics in hospital and house sewage - a pilot project for the city of Dresden." Pharmazie 73(5): 260-263.

Kosma, C. I., et al. (2010). "Occurrence and removal of PPCPs in municipal and hospital wastewaters in Greece." Journal of Hazardous Materials 179(1): 804-817.

Kümmerer, K., et al. (1997). "Biodegradability of the anti-tumour agent ifosfamide and its occurrence in hospital effluents and communal sewage." Water Research 31(11): 2705-2710.

Künnemeyer, J., et al. (2009). "Speciation analysis of gadolinium chelates in hospital effluents and wastewater treatment plant sewage by a novel HILIC/ICP-MS method." Environ Sci Technol 43(8): 2884-2890.

Lemas, D. J., et al. (2021). "Estimating drug consumption during a college sporting event from wastewater using liquid chromatography mass spectrometry." Sci Total Environ 764: 143963.

Lenz, K., et al. (2005). "Presence of cancerostatic platinum compounds in hospital wastewater and possible elimination by adsorption to activated sludge." Sci Total Environ 345(1-3): 141-152.

Lenz, K., et al. (2007). "Monitoring, removal and risk assessment of cytostatic drugs in hospital wastewater." Water Sci Technol 56(12): 141-149.

Li, A., et al. (2022). "Occurrence Characteristics and Ecological Risk Assessment of Organophosphorus Compounds in a Wastewater Treatment Plant and Upstream Enterprises." Water 14(23): 3942.

Li, Z. H., et al. (2022). "Hospital sewage treatment facilities witness the fighting against the COVID-19 pandemic." J Environ Manage 309: 114728.

Lien, L. T. Q., et al. (2016). "Antibiotics in Wastewater of a Rural and an Urban Hospital before and after Wastewater Treatment, and the Relationship with Antibiotic Use—A One Year Study from Vietnam." International Journal of Environmental Research and Public Health 13(6): 588.

Lin, A. Y., et al. (2014). "Ketamine and the metabolite norketamine: persistence and phototransformation toxicity in hospital wastewater and surface water." Water Res 53: 351-360.

Liu, X., et al. (2010). "Analysis of hormone antagonists in clinical and municipal wastewater by isotopic dilution liquid chromatography tandem mass spectrometry." Anal Bioanal Chem 396(8): 2977-2985.

Lopez-Herguedas, N., et al. (2023). "Suspect Screening of Chemicals in Hospital Wastewaters Using Effect-Directed Analysis Approach as Prioritization Strategy." Molecules 28(3).

Mahnik, S. N., et al. (2007). "Fate of 5-fluorouracil, doxorubicin, epirubicin, and daunorubicin in hospital wastewater and their elimination by activated sludge and treatment in a membrane-bio-reactor system." Chemosphere 66(1): 30-37.

Martins, A. F., et al. (2018). "Occurrence of polycyclic aromatic hydrocarbons in surface water and hospital wastewater." J Environ Sci Health A Tox Hazard Subst Environ Eng 53(6): 501-516.

Mayoudom, E. V. T., et al. (2018). "Identification and quantification of 19 pharmaceutical active compounds and metabolites in hospital wastewater in Cameroon using LC/QQQ and LC/Q-TOF." Environ Monit Assess 190(12): 723.

Mazzitelli, J. Y., et al. (2018). "Evaluation of psychiatric hospital wastewater toxicity: what is its impact on aquatic organisms?" Environ Sci Pollut Res Int 25(26): 26090-26102.

Mohapatra, S., et al. (2023). "Wastewater surveillance of SARS-CoV-2 and chemical markers in campus dormitories in an evolving COVID - 19 pandemic." J Hazard Mater 446: 130690.

Mullot, J. U., et al. (2009). "Development and validation of a sensitive and selective method using GC/MS-MS for quantification of 5-fluorouracil in hospital wastewater." Anal Bioanal Chem 394(8): 2203-2212.

Nagarnaik, P., et al. (2010). "Concentrations and mass loadings of cardiovascular pharmaceuticals in healthcare facility wastewaters." Journal of Environmental Monitoring 12(11): 2112-2119.

Nagarnaik, P., et al. (2011). "Source characterization of nervous system active pharmaceutical ingredients in healthcare facility wastewaters." J Environ Manage 92(3): 872-877.

Nagarnaik, P. M., et al. (2012). "Healthcare facility effluents as point sources of select pharmaceuticals to municipal wastewater." Water Environ Res 84(4): 339-345.

Nagarnaik, P. M., et al. (2010). "Concentrations and mass loadings of hormones, alkylphenols, and alkylphenol ethoxylates in healthcare facility wastewaters." Chemosphere 78(8): 1056-1062.

Najam, T. and I. Hashmi (2024). "Monitoring of university wastewater within the sewage system and its performance evaluation through integrated constructed wetlands." Environ Monit Assess 196(4): 403.

Nasri, E., et al. (2024). "Pharmaceuticals and personal care products in Tunisian hospital wastewater: occurrence and environmental risk." Environ Sci Pollut Res Int 31(2): 2716-2731.

Nasri, E., et al. (2017). "Cytotoxic effects of seven Tunisian hospital wastewaters on the proliferation of human breast cancer cell line MDA-231: correlation with their chemical characterization." Environ Sci Pollut Res Int 24(25): 20422-20428.

Negreira, N., et al. (2014). "Cytostatic drugs and metabolites in municipal and hospital wastewaters in Spain: filtration, occurrence, and environmental risk." Sci Total Environ 497-498: 68-77.

Ngigi, A. N., et al. (2019). "Occurrence of antibiotics residues in hospital wastewater, wastewater treatment plant, and in surface water in Nairobi County, Kenya." Environ Monit Assess 192(1): 18.

Nkansah, M. A., et al. (2016). "Characterization of Beauty Salon Wastewater from Kwame Nkrumah University of Science and Technology, Kumasi, Ghana, and Its Surrounding Communities." Environ Health Insights 10: 147-154.

Papageorgiou, M., et al. (2019). "Comprehensive investigation of a wide range of pharmaceuticals and personal care products in urban and hospital wastewaters in Greece." Sci Total Environ 694: 133565.

Pérez-Alvarez, I., et al. (2018). "Determination of metals and pharmaceutical compounds released in hospital wastewater from Toluca, Mexico, and evaluation of their toxic impact." Environ Pollut 240: 330-341.

Rawat, M., et al. (2003). "Inventory compilation and distribution of heavy metals in wastewater from small-scale industrial areas of Delhi, India." J Environ Monit 5(6): 906-912.

Rodríguez-Rodríguez, C. E., et al. (2023). "Occurrence and risk assessment of pharmaceuticals in hospital wastewater in Costa Rica." Chemosphere 339: 139746.

Sá, M. F. T., et al. (2022). "Tracking pollutants in a municipal sewage network impairing the operation of a wastewater treatment plant." Sci Total Environ 817: 152518.

Sakina, N., et al. (2023). "Heavy metals assessment of hospital wastewater during COVID-19 pandemic." International Journal of Public Health Science (IJPHS) 12: 187.

Sánchez-Avila, J., et al. (2009). "Determination and occurrence of phthalates, alkylphenols, bisphenol A, PBDEs, PCBs and PAHs in an industrial sewage grid discharging to a Municipal Wastewater Treatment Plant." Sci Total Environ 407(13): 4157-4167.

Santos, L. H., et al. (2013). "Contribution of hospital effluents to the load of pharmaceuticals in urban wastewaters: identification of ecologically relevant pharmaceuticals." Sci Total Environ 461-462: 302-316.

Saunders, L. J., et al. (2016). "Pharmaceutical concentrations in screened municipal wastewaters in Victoria, British Columbia: A comparison with prescription rates and predicted concentrations." Environ Toxicol Chem 35(4): 919-929.

Saussereau, E., et al. (2013). "Determination of levels of current drugs in hospital and urban wastewater." Bull Environ Contam Toxicol 91(2): 171-176.

Sharma, G., et al. (2022). "Direct injection green chromatographic method for simultaneous quantification of amoxicillin and amikacin in maternity hospital wastewater (Sagar, India)." Environ Pollut 296: 118719.

Sharma, P., et al. (2015). "Monitoring hospital wastewaters for their probable genotoxicity and mutagenicity." Environ Monit Assess 187(1): 4180.

Tania, K. A., et al. (2022). "Emerging small-scale textile industries in residential areas of Mirpur, Dhaka City, Bangladesh: an assessment of the discharged wastewater quality and potential impacts." Environ Monit Assess 194(8): 560.

Thomas, K. V., et al. (2007). "Source to sink tracking of selected human pharmaceuticals from two Oslo city hospitals and a wastewater treatment works." J Environ Monit 9(12): 1410-1418.

Ulvi, A., et al. (2022). "Fate of selected pharmaceuticals in hospital and municipal wastewater effluent: occurrence, removal, and environmental risk assessment." Environ Sci Pollut Res Int 29(50): 75609-75625.

Van Hoi, B., et al. (2021). "Determination of Pharmaceutical Residues by UPLC-MS/MS Method: Validation and Application on Surface Water and Hospital Wastewater." J Anal Methods Chem 2021: 6628285.

Vaudreuil, M. A., et al. (2022). "Pharmaceutical pollution of hospital effluents and municipal wastewaters of Eastern Canada." Sci Total Environ 846: 157353.

Verlicchi, P., et al. (2012). "Hospital effluent: Investigation of the concentrations and distribution of pharmaceuticals and environmental risk assessment." Science of The Total Environment 430: 109-118.

Verlicchi, P. and E. Zambello (2016). "Predicted and measured concentrations of pharmaceuticals in hospital effluents. Examination of the strengths and weaknesses of the two approaches through the analysis of a case study." Sci Total Environ 565: 82-94.

Vo, T.-D.-H., et al. (2016). "Investigation of antibiotics in health care wastewater in Ho Chi Minh City, Vietnam." Environmental Monitoring and Assessment 188(12): 686.

Wang, Z., et al. (2021). "Comparison of tobacco use in a university town and a nearby urban area in China by intensive analysis of wastewater over one year period." Water Res 206: 117733.

Wei, R., et al. (2012). "Occurrence of ciprofloxacin, enrofloxacin, and florfenicol in animal wastewater and water resources." J Environ Qual 41(5): 1481-1486.

Wei, R., et al. (2011). "Occurrence of veterinary antibiotics in animal wastewater and surface water around farms in Jiangsu Province, China." Chemosphere 82(10): 1408-1414.

Weissbrodt, D., et al. (2009). "Mass flows of X-ray contrast media and cytostatics in hospital wastewater." Environ Sci Technol 43(13): 4810-4817.

Yilmaz, G., et al. (2017). "Characterization and toxicity of hospital wastewaters in Turkey." Environ Monit Assess 189(2): 55.

Yuan, S., et al. (2013). "Detection, occurrence and fate of 22 psychiatric pharmaceuticals in psychiatric hospital and municipal wastewater treatment plants in Beijing, China." Chemosphere 90(10): 2520-2525.

# Graphic

Graphic in S1 Text: Increasing interest in WBE
